# Insights into the Conserved Regulatory Mechanisms of Human and Yeast Aging

**DOI:** 10.3390/biom10060882

**Published:** 2020-06-09

**Authors:** Rashmi Dahiya, Taj Mohammad, Mohamed F. Alajmi, Md. Tabish Rehman, Gulam Mustafa Hasan, Afzal Hussain, Md. Imtaiyaz Hassan

**Affiliations:** 1 Centre for Interdisciplinary Research in Basic Sciences, Jamia Millia Islamia, New Delhi 110025, India; taj144796@st.jmi.ac.in; 2 Department of Pharmacognosy, College of Pharmacy, King Saud University, Riyadh 11451, Saudi Arabia; malajmii@ksu.edu.sa (M.F.A.); m.tabish.rehman@gmail.com (M.T.R.); afzal.hussain.amu@gmail.com (A.H.); 3 Department of Biochemistry, College of Medicine, Prince Sattam Bin Abdulaziz University, P.O. Box 173, Al-Kharj 11942, Saudi Arabia; mgulam@gmail.com

**Keywords:** aging, evolutionary conservations, budding yeast, longevity, genomic instability, calorie restriction, loss of proteostasis, histone dynamics and drug discovery

## Abstract

Aging represents a significant biological process having strong associations with cancer, diabetes, and neurodegenerative and cardiovascular disorders, which leads to progressive loss of cellular functions and viability. Astonishingly, age-related disorders share several genetic and molecular mechanisms with the normal aging process. Over the last three decades, budding yeast *Saccharomyces cerevisiae* has emerged as a powerful yet simple model organism for aging research. Genetic approaches using yeast RLS have led to the identification of hundreds of genes impacting lifespan in higher eukaryotes. Numerous interventions to extend yeast lifespan showed an analogous outcome in multi-cellular eukaryotes like fruit flies, nematodes, rodents, and humans. We collected and analyzed a multitude of observations from published literature and provide the contribution of yeast in the understanding of aging hallmarks most applicable to humans. Here, we discuss key pathways and molecular mechanisms that underpin the evolutionarily conserved aging process and summarize the current understanding and clinical applicability of its trajectories. Gathering critical information on aging biology would pave the way for future investigation targeted at the discovery of aging interventions.

## 1. Introduction

Aging is a multifactorial, highly complex biological process that results in compromised physiological functions. Aging is manifested by a progressive decline in vitality and, in humans, greatly increases the susceptibility of numerous diseases, which include cancer and diabetes along with various metabolic, neurodegenerative, and cardiovascular disorders. Continued efforts and ongoing investigations in recent years have led to a unanimous understanding of the molecular basis of aging mechanisms. To delineate the conserved longevity pathways among all eukaryotes and in different experimental contexts, numerous strides have been made to efficiently categorize the molecular and cellular hallmarks of aging [1]. Invertebrate model systems are valuable tools to decipher the underlying mechanism of aging, mainly due to their short lifespan and ease of genetic manipulation. Fruit flies, nematodes, and budding yeast are the most frequently used model organisms in aging research [2,3,4,5].

Budding yeast provides an excellent system for the analysis of aging and has contributed immensely to the aging biology field over the last three decades. *S. cerevisiae* aging can be modelled in two ways: replicatively or chronologically. Replicative lifespan (RLS) is defined as the number of times a mother cell divides and produces a daughter cell before undergoing replicative senescence; however, chronological lifespan (CLS) is the length of time that yeast survives in the stationary phase following nutrient exhaustion [6]. Therefore, RLS gives information about the number of times a cell can divide, whereas, CLS provides an answer about how long a cell can stay alive without dividing. Phenotypes associated with RLS and CLS have been used to study the aging of stem cells (dividing) and post-mitotic cells (non-dividing), respectively. Here, we review the RLS mechanism of yeast cells and their association with humans.

RLS in yeast was demonstrated in 1959 and has been employed as a model for dividing cells in higher eukaryotes due to its accuracy in replicative assays. Interestingly, the longevity factors identified in yeast have been reported to modulate the aging mechanisms in invertebrate and mammalian models. Moreover, yeast aging studies have facilitated the identification and development of many of the best candidates for anti-aging drugs.

Numerous genetic, nutritional, and pharmacological interventions that increase yeast RLS have similar effects in higher eukaryotes. For instance, a variety of calorie restriction (CR) interventions has been reported for dramatic lifespan extension in a diverse range of organisms, including yeast, flies, worms, mice, rats, and rhesus monkeys [7,8]. The other highly studied longevity pathways in budding yeast include mTOR signaling and sirtuin activity [9,10,11]. Moreover, nine hallmarks described the time-dependent functional decline during the aging process: epigenetic alterations, genomic instability, mitochondrial dysfunction, telomere attrition, loss of proteostasis, cellular senescence, deregulated nutrient sensing, stem cell exhaustion, and altered intercellular communication [1].

In humans, the coexistence of several pathologies is driven by the multipolar effects of unhealthy or premature aging [12,13]. Neurodegenerative diseases like Alzheimer’s, frontotemporal dementia, and Parkinson’s are prevalent and occur among the age group of 45–64 [14,15,16] establishing that the continuum of broad neurodegenerative diseases is clinically, pathologically, and mechanistically associated with age with a spectrum of overlapping clinical symptoms [17,18,19,20].

Interestingly, human biological age can be determined with age-associated alterations in the metabolome, telomere biology, chromatin methylation, and transcriptional signatures [21,22], and the hallmarks of aging were found to be tightly linked, encompassing numerous biochemical alterations to affect metabolism. Many gene variants associated with long lifespan have been identified and were linked to metabolic control of cells [23]; conversely, pre-mature aging caused by genetic syndromes is associated with a strong link existing between genomic instability and severe metabolic defects [24]. Despite the genetic determination of progeroid diseases arising from either direct or indirect involvement of metabolism and genomic instability, the potential of conserved aging pathways is underscored.

**Figure 1:**
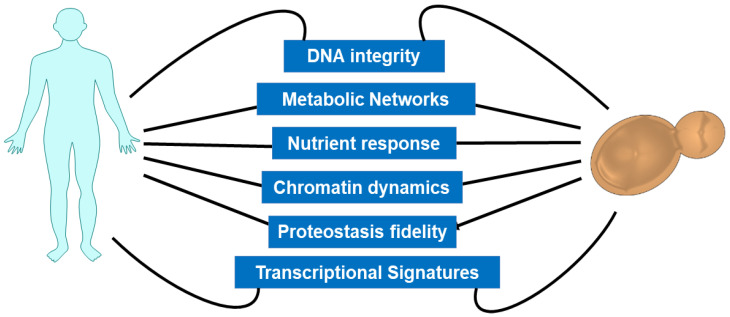
Conserved hallmarks of aging between yeast and humans. The conserved biological pathways known to modulate lifespan from yeast to higher eukaryotes are illustrated.

Here, we discuss our current understanding of the dynamic and conserved nature of aging-associated putative molecular mechanisms in divergent organisms with a focus on yeast and humans (Figure 1) and highlight the conserved longevity pathways and age-associated genes. We illustrate how aging affects the chromatin dynamics, molecular markers, and metabolic nodes and discuss aging interventions in modulations of cell growth and metabolism. Finally, we talk about a conceptual framework explaining the highly complex molecular nature of currently acknowledged aging hallmarks combining the accumulation of known, predictable, and unrecognized specific changes driven by the aging mechanism.

## 2. Integrative Biology of Yeast Aging: Pro-Longevity Interventions

The life of a yeast cell commences as a daughter cell separated from a mother cell, immediately after which, the daughter cell can produce its progeny. Each mother cell undergoes a limited number of cell divisions, which can be represented as an aging model for mitotic cells in higher eukaryotes. The RLS of budding yeast is a characteristic phenotype resulting from asymmetric cellular division, during which the mother cell retains a large volume compared to the daughter cell and a variety of factors are differentially partitioned between the mother and daughter cell [25]. Differential partitioning has been evolutionarily selected for retention of damaged material in the mother cell, providing maximum fitness for the growing daughter cells [26]. This asymmetric division leads to mother cell aging, while daughter cells retain a youthful phenotype. Essentially, the yeast cell division process can be compared with the aging hallmarks of stem cell exhaustion and cellular senescence in humans, indicating the decline in regenerative potential with aging [1,27].

Compelling shreds of evidence were observed for the involvement of various aging hallmarks in yeast RLS. For instance, genetic instability in yeast can be represented by the presence of self-replicating extrachromosomal ribosomal DNA circles (ERCs), formed from extensive homologous recombination (HR) between the multiple tandem rDNA repeats arranged as a 9.1-Kb unit at Chr.XII [28]. ERCs are the first reported “senescence factors”, an asymmetrically inherited form of molecular damage [28], and showed biased retention in the mother cell due to the septin-dependent lateral diffusion barrier between mother and daughter [29]. The presence of an origin of replication in ERCs leads to their replication and accumulation in S-phase [29], which are speculated to prevent further cell divisions through diluting the efficiency and fidelity of DNA replication machinery over time [28].

Many studies suggest that the natural decline in proteostasis pathways can have serious impacts on longevity. Yeast has been recognized as a paradigm to understand the general principles of protein maturation in the endoplasmic reticulum (ER) [30]. After attaining native conformations, proteins are sorted into ER-to-Golgi; however, failing to do so leads to an ER-associated degradation (ERAD) pathway which includes retrotranslocation of proteins into cytosol followed by their proteasomal degradation [30]. Insufficient degradation of proteins leads to the accumulation of misfolded aggregates which exerts ER stress through activation of an adaptive unfolded protein response (UPR) [31]. The UPR generates a homeostatic feedback loop demonstrated by *Ire1* or *Hac1* mutants. Resilient aggregates of proteins are found to increase in aged yeast cells. The defects in this normal pathway represent dysfunctions in protein homeostasis. For instance; loss of proteasomal subunits and chaperones drives yeast aging. Conserved subunits of proteostasis pathway share common functions across eukaryotes and play divergent roles in aging mechanisms in human pathologies.

Mitochondrial respiration defects accelerate aging through disturbances in protein homeostasis and lead to reactive oxygen species (ROS) production, which plays roles in metabolic dysfunction and numerous diseases. An increase in RLS requires active respiration; however, ROS dramatically affects yeast RLS. It was observed that RAS signaling plays a regulatory role in respiratory-deficient mutants [32]. In line with this, *yno1 Δ* mutant encoding an ER-localized NADPH oxidase dramatically decreases ROS production and RLS extension [32] (Table 1). Conversely, it was also suggested that the absence of a functional ETC (electron transport chain) or mtDNA potentially leads to RLS extension [33]. For instance, nucleus-encoded mitochondrial translation factor *sov1Δ* mutant leads to RLS extension mediated by Sir2 [34]. Therefore, an intricate balance within metabolic pathways modulates lifespan.

**Figure 2:**
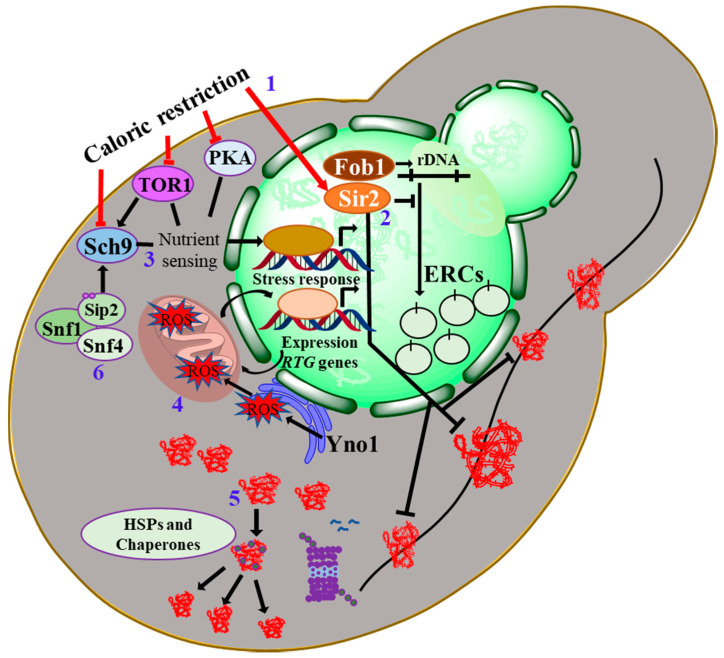
Pathways modulating the yeast lifecycle. Illustration of the major pathways that modulate yeast aging. (**1**) Calorie restriction mitigates the detrimental changes associated with aging. CR-mediated RLS extension is driven by both NAD^+^ salvage genes like SIR2 as well as Sir2-independent mechanisms modulated by reduced TOR/Sch9 and Ras-PKA signaling pathways, which play significant roles in growth metabolism and stress response. (**2**) A member of the sirtuins family, SIR2, an NAD^+^-dependent deacetylase-mediated aging pathway, represents the major player of yeast aging. SIR2 overexpression extends yeast RLS by suppressing ERCs formation through rDNA instability. SIR2 deletion leads to a shorter lifespan of daughter cells resulting from a defect in the asymmetric retention of oxidatively damaged proteins. (**3**) Nutrient response affects the aging pathways in many ways and regulates TOR signaling which is inhibited by nutrient stress. (**4**) Mitochondrial dysfunctions could initiate a retrograde response to regulate aging. Moreover, Yno1 modulates the accumulation of ROS. (**5**) The UPR mechanism helps in the removal of protein aggregates formed in response to the defective mechanism accompanying aging. HSPs and chaperone overexpression helps in the removal of damaged protein aggregates. (**6**) Sch9 can also be independently regulated by Snf1 through Sip2 acetylation, a component of the yeast Snf1 complex. These pathways are highly conserved and play similar roles in the modulation of the longevity of flies, worms, and mice.

*S. cerevisiae*, as well as all other eukaryotes, essentially require the local and global maintenance of chromatin structure for normal cellular functions through transcriptional activation or repression. Epigenetic alterations strongly influence lifespan. Relaxation of chromatin during aging is detrimental to genomic integrity and subsequently leads to aberrant expression of the genome. However, the reduction of certain histone modifications [35,36] and enhanced histone expression extends the lifespan [37]. Moreover, the expression of specific histone variants is associated with longevity; however, it remains to be demonstrated whether these changes are the cause or a consequence of aging. Yeast histone deacetylase Sir2 (silent information regulator) is highly conserved and extensively studied for its role in lifespan extension [38]. Age-related increase in H4K16 acetylation is reversed by the action of Sir2. Interestingly, transcriptional co-activators SAGA/SLIK subunit Sgf73 function as a deubiquitinase, which physically interacts with Sir2 and alters its deacetylation roles [39]. Conversely, *sgf73Δ* mutants showed increased telomeric silencing and are remarkably long-lived due to their increased Sir2 functions [39]. These roles, therefore, highlight hitherto unknown links of ubiquitination, rDNA silencing, and H4K16 acetylation. Figure 2 illustrates some of the pathways and genes, which potentially module yeast aging and play highly conserved roles in higher eukaryotes as well.

## 3. Epigenomic Landscape and Dynamics Underlying Aging

The epigenome consists of covalent and non-covalent modifications associated with DNA and accompanying histone proteins, which potentially control transcription, chromatin architecture, and genome stability [59]. The epigenome is very dynamic and can tremendously affect cell fate. The current hypothesis, which links aging with epigenomics, is the presence of distinct histone modifications, DNA methylation, loss of heterochromatin, and specific microRNA expressions. Budding yeast is ideal to gain insights into global genomic changes owing to its easily defined RLS. General loss of histones appears to be an emerging epigenetic trend that is conserved from yeast to humans. Evidence suggests that histone loss is strongly linked to cell division. In line with this, studies in both aged yeast and human fibroblasts using MNase digestion demonstrated ~50% reduction in nucleosomal density due to profound histone loss correlated with open chromatin state [60,61]. The deletion of histone chaperone Asf1 or H3 acetylation of K56 results in an increase in nucleosome spacing and a shorter lifespan in yeast [37]. Moreover, the fuzziness score distribution of nucleosomal contents indicates that nucleosomes commonly become “fuzzier” during aging [60]. Aging results in global alterations of nucleosome spacing and positioning and lead to reduced periodicity to exhibit gene-specific transcriptional defects. Ectopic expression of histones could reverse aging through rigid chromatin reorganization and global gene silencing [37]. Aging-associated nucleosomal repositioning and varying transcriptional outputs underlie dynamic histone modifications that have been extensively studied in *S. cerevisiae, C. elegans, and M. Musculus* [35,62,63]. Moreover, significant differences in histone modification landscape have been identified in aged human nucleosomes [64].

Several relevant studies have set the platform of chromatin foundation for modulating lifespan. These chromatin alterations include post-translational modifications such as reversible histone methylation and acetylation. H3K4 methylation represents a transcriptionally active mark. Studies from *C. elegans* showed that the reduction of proteins that catalyze the histone methylation (Trithorax proteins: SET2, WDR-5, and Ash-2) reaction increases lifespan, suggesting detrimental effects of H3K4me in the context of aging [36]. H3K27me3 is a repressive histone mark that is directly correlated with aging and its increase through the loss of demethylase UTX-1 resulted in a 30% lifespan extension in *C. elegans* [65]. Interestingly, overactivation of KDM6A (human homolog of UTX-1) promotes aging in both human and macaque tissues, suggesting a conserved aging regulatory mechanism of this histone mark through modulation of insulin receptor *IGF1R* [65]. In drosophila too, H3K27me3 regulates lifespan suggesting a highly conserved role [66]. Changes in the bivalent domains characteristically represented by the presence of both activating and repressive H3K4me3 and H3K27me marks in mammalian mesenchymal stem cells has been linked to adipocyte differentiation and aging [67]. Increased H3K27me3 has been observed at TSS of ~30% genes in aged mice suggesting a general increase of transcription silencing and heterochromatin with age [68]. An increase in lifespan is perhaps linked with global alterations in the transcriptional profiles of longevity-associated genes.

In both yeast and humans, dimethylation of H4K20 and H3K79 on DNA lesions is required for initiation of DDR through 53BP1 recruitment, and its reduction potentially accelerates the aging [69,70]. Cultured cells from Hutchinson–Gilford progeria syndrome (HGPS) patients showed an increased level of H4K20me marks and lowered levels of H3K9 and H3K27 methylation marks [71]. Moreover, loss of H4K20me3 marks in HGPS patients was specifically observed in gene-poor heterochromatin regions, further confirming the conserved roles of heterochromatin silencing in accelerated aging. Additionally, modulation of H4K20me3 levels was also observed in tissues of the kidney and liver of aged rats [72]. Similar patterns of loss of H3K9me were observed in drosophila, supporting the notion of accessible open chromatin with aging.

DNA methylation is closely linked to aging, and there are a marked decline in 5mC (5-methyl cytosine) content in aged cells of mice as well as humans [73]. Interestingly, the levels of 5mC are reported to decline with aging [74]. The reduction in 5mC levels could be attributed to the decreased levels of DNA-methyltransferases (DNMTs) and folic acid with aging. In agreement, a marked decline of global 5mC has also been observed in several age-dependent neurodegenerative and cardiovascular disorders and cancer [75]. In addition to the global reduction of 5mC, some genomic areas represent hypermethylation during aging [76] preferentially localized on bivalent chromatins [77]. However, site-specific hypermethylation and its role in aging are still obscure. Few reports suggested that Polycomb-targeted chromatin regions tend to be hypermethylated during aging [78].

The crucial role of 5mC in multiple cellular functions has driven researchers to focus on its contribution to aging. The association of methylation patterns on disease-related genes has provided significant insights into their specific roles. This includes altered methylation patterns in Alzheimer’s susceptibility gene TREM2 [79], and in PPARGC1A mRNA in diabetes [80]. Despite its link to aging, distinct methylation patterns likely function as an adaptive response towards stress, however, the age-associated conserved CpG sites with additional biological roles remain to be determined.

## 4. Histone Acetylation and Sir2 Roles in Aging

Histone acetylation is associated with actively transcribed chromatin, and its global decline is associated with aging in human diploid fibroblasts [81] and aged rodent brains [82]. Sirtuins are the members of the conserved family of NAD^+^-dependent histone deacetylases that regulate key metabolic pathways, such as cell survival, metabolism and proliferation, DNA repair, cell senescence, apoptosis, and calorie restriction [83]. Four *SIR* genes—SIR1, SIR2, SIR3, and SIR4—were first identified in yeast as essential components for the transcriptional repression of silent mating-type loci, HML and HMR. Defects in *SIR* expression resulted in sterility in haploid strains. Sir2p functions as a silencing factor in SIR holo-complex, a transcriptional silencing multiprotein complex [84], and its role in aging has been discovered thereafter. *SIR2* homologs were identified in organisms ranging from bacteria to humans [83]. Sir2 reinforced heterochromatin silencing through compact chromatin structure and potentially increased lifespan, whereas *sir2Δ* resulted in hyper-recombination, genomic instability, loss of transcriptional silencing, shortening of telomere and loss of positional effect variegation (PEV) [41]. Conversely, *SIR2* overexpression drastically extends the lifespan [41]. Immunofluorescence studies indicate Sir2 is majorly localized in the nucleolus [85] and facilitates heterochromatin silencing of mating-type loci and telomeres in yeast. Moreover, overexpression of Sir2 orthologs was reported for extension of lifespan in both worms [86] and flies [87] mediated by insulin-like signaling pathway and CR respectively. The independent studies performed in non-mammalian model systems established Sir2 activation as the first genetic intervention able to promote longevity.

Sir2 deacetylates histone H4K16ac, H3K9ac, and H4K8ac marks with a preference for H4K16 [88], and Sir2-mediated deacetylation of H4K16ac promotes high-affinity binding of the SIR holo-complex [89]. Interestingly, H4K16ac is defined as a mark of gene activation, and its levels were found to be decreased in mouse models of HGPS [90]. In yeast, H4K16ac levels directly correlate with age and conversely *sas2* (H4K16 HAT) mutant showed lifespan extension. The spreading of the transcriptionally silenced region is controlled by the dynamic and opposing activity of several HATs, most importantly Sas2, which antagonizes Sir2 function through H4K16 acetylation and establishes the telomeric and euchromatic boundaries. Sas2–Sir2 actions generate a gradient of H4K16ac and deacetylation [91]. Not surprisingly, *sas2Δ* extends yeast lifespan and modulates chromatin changes in old cells [35]. Multiple Rap1p binding sites located at the terminal telomeric repeats recruit Sir2 and Sir4 proteins as a complex with Rap1 protein for telomeric silencing. Sir2/Sir4 complex recruitment activates the deacetylation of histones and triggers the spreading of telomeric chromatin, together with Sir3 across the sub-telomeric regions, typically up to 1–2 kb [92].

Sir2p recruitment to the rDNA locus occurs differently compared to HML, HMR, and telomeres and requires Net1p (nucleolar silencing establishing factor and telophase regulator 1) as a part of the RENT complex (regulator of nucleolar silencing and telophase exit) [93], responsible for the establishment, maintenance and spreading of heterochromatin. In yeast, H3K56ac is essentially deposited by the activities of Asf1 and HAT Rtt109. Mutations in any of these genes reduce H3K56ac levels as well as histone abundance. Interestingly, aged yeast cells showed substantial global depletion of H3K56ac, which significantly decrease RLS. Consistent with what was observed in yeast, H3K56ac levels decrease in human cells in vitro with age. The balance of acetylation marks is essential to maintain a normal healthy lifespan. The global changes in histone modifications could potentially influence many hitherto-unidentified age-related transcriptional outputs. Apart from these mechanisms, understanding of the functional roles of other modifications, such as ubiquitylation and SUMOylation, is incomplete due to the multitudes of their targeted substrates. The mammalian sirtuin (SIRT) family, comprised of seven members (SIRT 1-7) out of which three (SIRT1, SIRT2, and SIRT3) deacetylate H4K16 [94]. The reduction of SIRT activity promotes cellular aging through increased H4K16ac. SIRT1 (Sir2 homolog) was reported for its protective functions in several diseases in murine models, including cancers, cardiovascular disease, metabolic disorders, neurodegeneration, and aging [95], and its loss is implicated in aging and its associated disorders [96,97]. Additionally, Sirt6 also regulates lifespan through H3K9 deacetylation, and Sirt6 knock-out mice show genomic instability, including telomeric fusions, and an aging-like phenotype [98,99]. Sirtuins require NAD+ for their functions; therefore, the decline in cellular NAD+ levels during accelerated aging reduces the activity of sirtuins [100]. Recently, Sir2 mutations have been characterized using yeast genetic screen, which allows Sir2 functions with increased enzymatic activity at low NAD+ levels leading to extended RLS. Conserved residue mutations, K475E, and K475Q improved the catalytic efficiency of Sir2 as well as hSIRT1, possibly through conformational changes, and evolved in yeast to persist in low-NAD+ settings [101].

**Figure 3:**
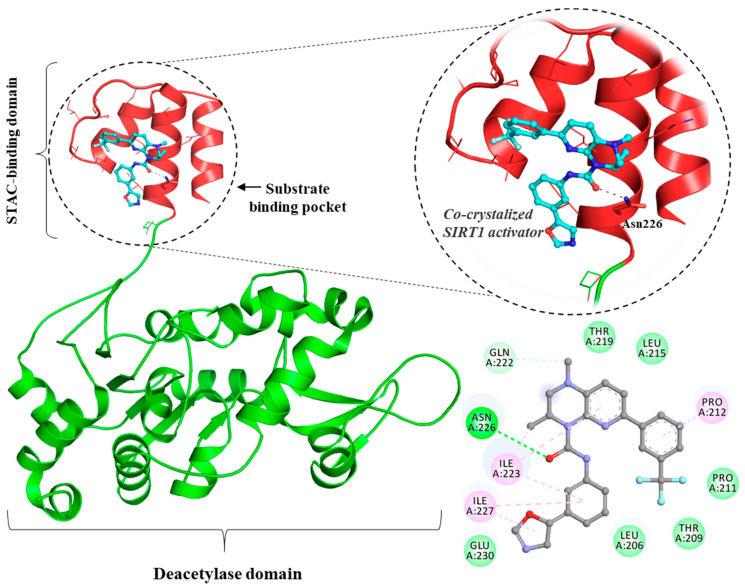
Structural representation of SIRT1–activator complex (PDB ID: 4ZZJ, Ref. 106). Right lower panel showing 2D representation of activator (3S)-1,3-dimethyl-N-[3-(1,3-oxazol-5-yl)phenyl]-6-[3-(trifluoromethyl) phenyl]-2,3-dihydropyrido [2,3-b]pyrazine-4(1H)-carboxamide and its interactions to SIRT1 binding-pocket residues. (Dark green shows conventional hydrogen bonds, and other residues participate in different significant interactions.)

Modulation of sirtuin functions is a critical step in aging pathways throughout eukaryotes and could be regulated by pharmacological interventions. Overexpression and catalytic activation of SIRT1 in mice and the use of small sirtuin-activating compounds (STACs: resveratrol and SRT1720), has been reported to increase lifespan through boosting insulin sensitivity and providing a guard against mitochondrial impairments and oxidative damage [102]. Pharmacological interventions for increasing SIRT1 activity have been reported to delay aging, and associated disorders such as obesity, inflammation, cancer, autoimmune, neurodegenerative, metabolic, and cardiovascular diseases [103,104]. STACs improve cellular metabolism and confer protection against biological disorders in old age. Owing to their promising activities, some of the STACs are in clinical trials for the treatment of various disorders [105]. Understanding of the molecular interface which governs STAC–SIRT1 interactions holds significant promise as a therapeutic intervention for several human diseases [106] (Figure 3). Asn226 located in the STAC binding domain is a significant residue that forms an H-bond with the activator [106]. Glu230 in SIRT1, located in a structurally stable N-terminal domain, was reported to be critical for SIRT1 activation by reported STAC scaffolds. Indeed, SIRT1 activation is attenuated by the substitution mutations at position 230 [107]. Analysis of the activator–protein interaction surface has identified many potential residues (Figure 3).

## 5. Associating Aging with Genomic Instability

DNA integrity is frequently challenged by the agents reacting with DNA, and the buildup of lesions frequently causes mutations, transcription, and replication block, which subsequently triggers the DNA Damage Response (DDR). The DDR leads to cell cycle arrest and activation of signaling pathways underlying DNA repair, apoptosis, or cellular senescence. Genomic instability is implicated as a causal factor in aging in model organisms and humans [108]. Inadvertent alterations within the genome are hypothesized to be central to aging. In humans, a high incidence of cancer represents a striking link with increasing age. Indeed, genomic instability is represented as a hallmark of cancer and has been identified in all organisms. Genomic instability in yeast has been extensively studied due to its significant connections to cancer [109]. Aging yeast cells switch to a condition of high genomic instability during late RLS and persist in that state until they die. Moreover, genome alterations taking place due to defects in DNA repair machinery accelerate aging. For instance, in humans, the clinical phenotype of Werner and Bloom syndrome caused by a mutation in *WRN*, the gene encoding RecQ DNA helicase, has received the title of an aging hallmark due to genomic instability. Functional WRN rescues telomeres stability and replication stress, and its loss leads to various age-related disorders like arteriosclerosis, osteoporosis, premature greying of hair, diabetes, growth retardation, and cancer [110]. Interestingly, SGS1 in yeast is the homolog of *WRN* and dramatically reduced the lifespan when deleted [48].

ERCC1–XPF-(Excision Repair Cross Complementation Group 1) is a DNA endonuclease complex required for NER, repair of DSBs, and ICLs (inter-strand crosslinks). Mutations within ERCC1 or XPF cause increased risk of cancer, XFE progeroid syndrome, xeroderma pigmentosum (XP), or cerebro-oculo-facio-skeletal syndromes characterized by accelerated aging along with developmental abnormalities [111]. Moreover, ERCC4 mutations, encoding one of the catalytic subunits of ERCC1-XPF, cause XFE progeroid syndrome [112] and Fanconi anemia [113], whose patients represent the symptoms of premature aging. ERCC4 and ERCC1 are the yeast homologs of RAD1 and RAD10 respectively, and function in the DDR pathways. Similarly, a mutation in RAD1 and RAD10 causes genomic instability in yeast clonal cultures [114]. Not surprisingly, the deletion of DDR pathway genes was reported to decrease the lifespan of yeast [115]. Transcriptional signatures of DNA damage have also been described in aging yeast, pointing at the occurrence of genome insults [116]. The presence of genomic instability in rDNA locus generating ERCs is a more relevant interpretation of aging in yeast. Indeed, reduction in the formation of ERCs results in a clear influence on aging observed with increased lifespan; conversely, an artificial increase in ERC formation resulted in a shorter lifespan [28].

Ribosomal DNA (rDNA) in humans consists of ~600 rDNA repeats, which encompass five chromosomes (Chr.1, 13, 14, 15, 21, and 22), and are identified as a highly transcribed region consistent with the abundance of ribosomal RNA/protein in cells [117]. Ribosome biogenesis occurs in both nucleolus and cytoplasm and is tightly regulated through an integrated feedback network based on nutrient availability. Due to their repetitive nature, rDNA sequences are prone to recombination and pose a potential threat to genomic integrity. Transcriptional levels of rDNA are maintained to ensure proper cellular homeostasis with ~1/3 rDNA repeats epigenetically silenced [118]. Interestingly, instability within rDNA repeats can predispose to cancer, Werner and Bloom syndrome, ataxia-telangiectasia, neurodegeneration, and premature aging. Moreover, cohesinopathies like Roberts Syndrome and Cornelia de Lange syndrome are also associated with alterations in the nucleolus [119,120]. However, whether these clinical outcomes are causes or consequences of rDNA instability or overall genomic DNA maintenance remains to be elucidated.

DNA replication stress induces high levels of genomic instabilities, including duplications, deletions, translocations, and aneuploidy within aging cells. Indeed, recent observations from yeast have identified the reduction of two replicative DNA polymerases—Polα and Polδ—resulting in a significant increase in chromosome deletions, duplications, rearrangements, and mitotic recombination [121]. Moreover, many human homologous genes were identified in yeast as having roles in genetic instability, cancer progression, and accelerated aging [122]. For instance, mandibular hypoplasia, deafness, progeroid features, and lipodystrophy (MDPL) syndrome is a Werner-like progeroid syndrome characterized by osteoporosis, loss of hearing, early onset of diabetes, lipodystrophy, accelerated aging and steatosis [123]. Ruijs–Aalfs syndrome (RAS) is also characterized as a Werner-like progeroid syndrome, and its patients have grey hair, osteoporosis, diabetes, atherosclerosis, cataracts, sarcopenia, and cancer [123]. Interestingly, MDPL and RAS are caused by mutations within the replicative polymerase POLD1 and SPRTN, which involves the repair of protein–DNA cross-links [123].

## 6. Calorie Restriction as an Anti-Aging Intervention: Molecular Mechanisms and Multitude Effects

**Figure 4:**
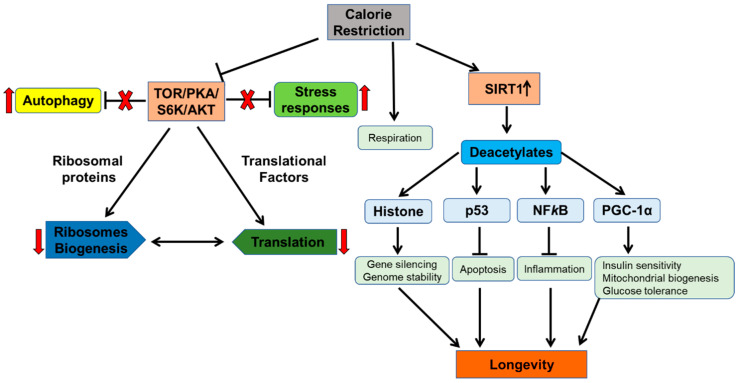
Schematic representation of pathways associated with caloric restriction and their downstream effects. The figure represents the conserved underlying response of caloric restriction and how this modulates aging and associated pathways. CR inhibits TOR functions, which result in decreased S6K activity and decreased protein translation, along with increased autophagy. Decreased ribosome biogenesis and translation then inhibits angiogenesis and cell-cycle progression. CR promotes SIRT1 activity which results in the deacetylation of many downstream targets which maintain heterochromatin, establish genomic stability, prevent the expression of pro-inflammatory genes, and decrease cell growth and proliferation, ultimately promoting longevity.

CR, defined as a dietary regimen with reduced intake of calories without acquiring malnutrition or lessening of essential nutrients [124], represents the most successful intervention for lifespan extension in yeast, worms, flies, and mammals. In yeast, CR is mediated by glucose reduction in growth media from 2 to 0.5% or lower, which leads to a dramatic lifespan increase of up to 30–40% [125]. CR decreases the uptake of nutrients, which ultimately leads to an imbalance in metabolic systems; conversely, to overcome this imbalance, cells initiate various evolutionary conserved signal transductions pathways to achieve equilibrium. In response to the reduction in calories, cells activate efficient metabolic pathways as well as the activation of remodeling mechanisms conferring defense against cell damage, whereas less efficient metabolic and synthetic pathways are blocked. This equilibrium mechanism responds to growth-associated pathways, such as insulin signaling (IGF-1), Target of Rapamycin (TOR), AMP-dependent kinases, improvement of mitochondrial redox, and regulation of autophagy (Figure 4). Autophagy (self-eating) constitutes a major protein turnover pathway that significantly declines with age. The nutrient restriction is a major stimulus for autophagy induction [126].

CR delays aging progression and the development of the age-related chronic disease. Evidence that CR significantly extends lifespan and retards aging was first observed in rats [127] and later documented in yeast [128,129] and Rhesus monkeys [130]. In yeast, CR enhances mitochondrial functions and leads to increased autophagy and resistance to diverse forms of stress along with a reduction in mRNA translation [131] (Figure 4).

Initial observation suggested that CR leads to a metabolic shift to augment NAD^+^ levels, which act as a substrate for Sir2-dependent deacetylation [128,129]. CR and Sir2 function in diverse genetic pathways in longevity promotion. For instance, CR increases the lifespan of a fork block 1 (*fob1Δ)* mutant; however, SIR2 overexpression fails to further extend the lifespan of *fob1Δ* strain [132], which suggests the presence of an independent pathway regulated by Sir2 through which CR affects longevity. Moreover, mutations within the genes of glucose-sensing pathways mimic the anti-aging effects of CR by inactivating nutrient responsive kinases, such as TOR, PKA, and Sch9 [53].

With the significant increase of research accomplishments from yeast, information on the CR effects in human and animal models have accumulated. In humans, data from inspection and randomized clinical trials showed that CR affects the same metabolic and molecular adjustments which are reported to improve health by decreasing molecular damage for longevity. For instance, CR restructures various metabolic factors implicated in cardiovascular diseases, diabetes, and cancer, identified as the leading basis of morbidity and mortality [133]. CR represents a non-pharmacological (physiological) intervention for aging. The effects of pharmacological intervention on the modulation of age comes from cell-line systems and mice models. Pharmacological modulation of aging can be observed by the small-molecular inhibitors against TOR and S6K1 in alleviating age-related disorders like aberrant insulin signaling and cancer [134,135]. Moreover, ongoing longitudinal studies from the NIH-funded CALERIE (Comprehensive Assessment of Long-term Effects of Reducing Intake of Energy) project, started more than a decade ago, represent information from randomized clinical trials of the effects of CR on human health [136]. Figure 4 represents many conserved aspects of calorie restriction which are reported to positively regulate aging and its associated pathways. In conclusion, many pathways regulate each other in distinct organisms to activate an evolutionarily conserved response through CR, which avoids energy expenditure on futile cellular pathways and redirects the energy for survival.

### 6.1. Signalling Nexus of Protein kinase-A in Cellular Aging

Numerous signaling pathways coordinate the intricate equilibrium between cellular growth and survival in response to extra-and intracellular stimuli. cAMP-dependent PKA pathway represents the key signaling node in yeast, responding primarily to the levels of glucose and fermentable sugars [137]. Glucose addition triggers a transient burst in the cAMP level which sets off a PKA-mediated phosphorylation cascade and transcriptional reprogramming. Cellular signaling mediated by PKA suppresses stress response and plays a prominent role in the transition of carbon availability. Interestingly, artificial over-stimulation of PKA during severe carbon starvation in the stationary phase recapitulates the glucose-derived transcriptional response and shortens lifespan; however, PKA mutations extend viability. In yeast, the induction of cAMP signaling requires the interaction of G alpha-protein Gpa2 and G-protein coupled receptor (GPCR) Gpr1 to activate adenylate cyclase (Cyr1), which in turn stimulates cAMP levels. PKA activation via cAMP then modulates growth, metabolism, and stress response. Cyr1 activation is highly dependent on Ras activity encoded by two Ras GTPase proteins: Ras1 and Ras2 in yeast. Ras activation is dependent on Cdc25 and Sdc25 [138]. The absence of Ras or Cdc25 leads to the abrogation of Cyr1 activity, which results in cAMP depletion, ultimately causing growth arrest and perpetual entry into the stationary phase G0.

To elucidate the effects of CR in yeast, various experiments were conducted to determine the lifespan extension by limiting glucose. The entry of glucose in cells is mediated by highly regulated hexose transporters (HXTP) and phosphorylation of glucose to glucose-6-phosphate occurs by hexokinases (Hxk1, Hxk2, and Glk1). Interestingly, *hxk2Δ* mutant strains have significantly extended lifespans [53]. Furthermore, it was also established that restricting the flow through the cAMP/PKA pathway by mutations within its component could mimic the lifespan extension reported with CR. Indeed, *gpr1Δ* and *gpa2Δ* genetically mimic CR through the reduction of PKA signaling [53] and exhibit an extended lifespan that fails to extend further with CR (Table 1). Moreover, *gpr1Δ* and *gpa2Δ* mutant strains further lead to a longer lifespan of *sir2Δ* and *fob1Δ* yeast strains [132]. Mutations in *CYR1/CDC35*, *CDC25,* and *TPK* genes reduce the PKA activity and extend lifespan, whereas *PDE2* mutation increases PKA activity and shortens life-span [139]. The *cdc25-10* mutant functions independently of the Msn2/4 stress-responsive TFs, which are negatively regulated by the PKA pathway and lead to the extended lifespan of yeast, suggesting that low glucose and low cAMP/PKA activity functions in similar pathways for life-span extension [139]. Conversely, activation of PKA decreases RLS significantly through disrupting Bcy1 (a negative regulator of PKA), exogenous cAMP addition, or CYR1 overexpression.

PKA has been identified as a potential inhibitory target for aging intervention. In humans, targeting of the cAMP/PKA pathway has gathered significant attention as an anti-aging intervention. Disruption of PKA-pathways promotes longevity and resistance to stress-induced cardiomyopathy in mice [140]. Analysis of the interaction between PKA and one of its inhibitors showed important residues required for potent inhibition (Figure S1) [141]. This information can be used to develop and characterize non-toxic compounds against PKA for maintaining good health with increasing age. Altogether, the significance of the cAMP/PKA pathway has been demonstrated in the regulation of the lifespan of yeast as well as higher eukaryotes, providing a rationale for the development of PKA inhibitors for delaying or preventing aging and its associated disorders.

### 6.2. The Target of Rapamycin and Sch9

The target of rapamycin (TOR) is conserved nutrient-sensitive S/T kinase identified as a major controller of cell growth and target of an immunosuppressive drug, rapamycin. *S. cerevisiae* has prominently contributed to the discovery of TOR and its roles in cell metabolism, stress, autophagy, ribosome biogenesis, translation, and actin organization [142]. Tor1 functions in lifespan extension were identified in a genetic screen to obtain long-lived mutant yeast strains. Interestingly, most long-lived strains were implicated in the TOR signaling pathway. Indeed, *tor1Δ* mutants have an extended RLS. Out of many identified substrates, Sch9 kinase is the best-characterized target of TOR [143]. Upon rapamycin treatment, rapid dephosphorylation of Sch9 occurs. Interestingly, glucose limitation (0.5 or 0.05% vs. 2%) fails to further extend the lifespan of *tor1Δ* and *sch9Δ* yeast mutants, indicating that the calorie restriction facilitated by limited glucose is mediated by the downregulation of TOR and SCH9 pathways [9]. The inhibition of TOR signaling was also reported in lifespan extension in worms [144], flies [145], and mice [146,147].

Clinically, TOR is implicated in numerous diseases. In mammals, two distinct multiprotein complexes, mTORC1 and mTORC2, are formed by mTOR kinases. Gene expression analysis of mTOR signaling identified interesting associations with longevity in humans [148]. Insulin and IGF1 significantly activate mTORC1, which further promotes translation via ribosomal protein S6K1 (mammalian homolog of Sch9), synthesis of fatty acids via sterol regulatory element-binding protein (SREBP) and differentiation of adipocytes via peroxisome proliferator-activated receptor gamma (PPARγ) and suppresses autophagy and biosynthesis of lysosomes via transcription factor EB (TFEB) [148]. Interestingly, activation of mTOR-PIK3CA-AKT is frequently observed in many human cancers, leading to the development of various small-molecule inhibitors targeting diverse nodes in these pathways.

Inhibitors for the ATP-binding pocket of mTOR are known as mTOR kinase domain inhibitors (TORKi) (Figure S2). First-generation and second-generation mTOR-inhibitors, rapalogs, and TORKi, respectively, are currently in clinical trials [149]. Structural aspects identified from mTOR-complex revealed an avidity-based approach to tackle mutations conferring drug-resistance in either the kinase or the FRB domain via the juxtaposition of the rapamycin–FRB-binding element-and TORKi-binding sites. Bivalent mTOR inhibitor could potentially inhibit mutant TOR class and provide high-affinity recognition [134].

Pharmacological inhibition of S6K1 kinase alleviates age-related disorders like aberrant insulin signaling and cancer [135,150]. Structural analysis of S6K1 with the inhibitor revealed many significant residues that can be targeted for pathologies associated with aging (Figure S3). The lifespans of yeast mutants *rpl10Δ, rpl31aΔ,* and *rpl6bΔ*, regulated by the TOR pathway, were found to be increased [9]. Previous reports identified the functional interaction of Rpl10 with the small 40S subunit protein Rps6 [151]. Indeed, alteration in the gene dosage of 60S RPL10 and 40S RPS6 subunits led to the alteration in the translational capacity of the cell, and a significant increase in lifespan [42]. Rps6 is also a target of S6 kinase (Sch9). Interestingly, it was observed that RLS extension by depleting 60S ribosomal subunits is mediated by Gcn4, a nutrient-responsive transcriptional activator [152]. Apart from these, a long-lived deletion mutant of *AFO1/MRPL25* encoding a subunit of the large subunit of the mitochondrial ribosome has been isolated, which confers an increase of 60% of yeast RLS [153]. Together, these reports suggested that a conserved TOR pathway modulates cell growth metabolism and functions in the regulation of longevity in organisms ranging from yeast to humans. Effectors and molecular targets of this pathway cooperate to regulate the lifespan across species, and their pharmacological targeting could potentially modulate age-associated disorders.

## 7. Loss of Proteostasis Networks in Aging

Proteostasis occurs through the regulation of protein production, degradation, autophagy, and protein folding mediated by chaperones. Aging compromises the cellular ability to preserve proteostasis and its loss aggravates several human pathologies, such as Alzheimer’s, Huntington’s, or Parkinson’s disease [154]. These proteinopathies are age-related disorders favored by the deterioration of proteostasis networks. The main components for proteostasis maintenance are chaperones and two proteolytic systems, autophagy and proteasome, which both collectively decide the protein turnover.

Autophagy represents a major protein turnover pathway that can maintain cellular homeostasis. Interestingly, autophagy and proteasome activity both decline with age [155,156], and abrogating the proteotoxic load extends lifespan. Autophagy failure has been associated with aging-related diseases like neurodegeneration or cancer [157]. Conversely, maintenance of an accurate autophagic activity contributes to lifespan extension. Activation of proteasome or autophagy via overexpression of proteasome components of autophagy regulators confers stress resistance and increases lifespan in *S. cerevisiae*, *C. elegans*, and *D. melanogaster* proteasome activation, and promotes lifespan extension and resistance to proteotoxicity [158,159]. Research on such interventions has recently emerged in mammals. Interestingly, inducing the expression of the autophagy-related protein ATG5 showed about a 20% increase in mouse lifespan and anti-aging phenotypes [160]. Indeed, most aging interventions for improving proteostasis demonstrate activation of autophagic properties. For instance, CR, rapamycin, resveratrol, and metformin directly act on autophagy and extend lifespan [158]. Interestingly, the upregulation of proteostasis through proteasome activation compared to chaperone has been documented to show dramatic effects on yeast lifespan [161] reflecting a need for increased proteostasis with age. The other dimensions of these studies would be to understand the roles of poorly explored “protein-repair” enzymes and their declining functions with aging. Interestingly, in yeast, protein damage due to oxidation has been reported to occur during aging with increased carbonyl levels [162]; moreover, in flies, overexpression of methionine sulfoxide reductase, which facilitates the reduction of oxidizes methionine, provides support for the value of the manipulation of repair enzymes as an anti-aging intervention.

The different conformational changes that occur in proteins are assisted by chaperones. Depending on the cellular availability of ATPs and chaperones, the final commitment of refolding or degradation is followed [163]. Poor cellular energetic with aging potentially alters chaperoning activities. For instance, lower ATP availability with age could repress chaperone activities, and undesired age-related modifications in the substrate protein can also interfere with the chaperone’s ability to recognize its target [163]. For example, the presence of glycated protein products interferes with chaperone functions. Conversely, the induction of ATP-independent chaperones has been recently identified in the aging brain [164]. Hsp70 and Hsp90 maintain proteome balance under normal conditions, and studies of *C. elegans* highlight that small HSPs form protective aggregates by trapping excess cytosolic proteins [165].

Stoichiometric loss in many protein complexes also occurs with aging. Indeed, the nuclear pore complex (NPC) loses stoichiometry and a substantial decrease in the levels of NPC components Nup116 and Nsp1 with age [166]. Reorganization of the disproportion within protein complexes naturally occurring with aging through enhanced proteasome activity could represent a significant mechanism employed by cells to minimize age-related phenotypes. Additional work would be vital to understand the underlying mechanism to increase lifespan through upregulation in proteasome activity as a potent modulator and hallmark of lifespan.

In yeast, CR leads to the induction of NQR1 expression, which is identified as a cytochrome b5 reductase localized to the plasma membrane. NQR1 overexpression in yeast sufficiently extends the lifespan (both RLS and CLS) mediated by CR by promoting respiratory metabolism, perhaps through moderating the NAD^+^/NADH balance. However, the effect of NQR1 overexpression on longevity was drastically reduced in *sir2Δ* strains, indicating an NQR1-SIR2 circuit for lifespan extension [167]. CR or fasting leads to the rise in NAD^+^ levels and SIRT1 function [168]. Interestingly, in humans, it has been reported that NQO1 (homolog of NQR1) regulates SIRT1 activity through the modulation of NAD^+,^ and, reversibly, SIRT1 leads to the induction of NQO1 (NAD(P)H Dehydrogenase Quinone 1) on both transcriptional and protein levels. Moreover, the physical interaction of NQO1 with SIRT1 was reported which was absent in the enzymatically dead SIRT1 H363Y mutant [169]. NQO1 also regulates ubiquitin-independent proteasomal degradation through its association with the 20S proteasome [170] and it acts as a gatekeeper to regulate the stability of several intrinsically disordered proteins (IDPs)—for instance, p53, p73, and PGC1α—in an NADH-dependent manner [171]. These data reinforce that conserved circuitry exists between yeast and humans and functions beneath CR.

## 8. Aging and Telomeres Dysfunction

Telomeres are simple tandem repeats at chromosome ends that protect the chromosomes and maintain genome integrity. Telomeres constitute a vast area of research in the field of aging, senescence as well as diseases. DNA replication on telomeres of eukaryotic linear chromosomes requires the RNA primer as a template to initiate the DNA synthesis which poses the “end-replication problem”. This inability of eukaryotic cells leads to telomere shortening at every S-phase of the cell cycle. Analysis of telomere lengths in cell-lines originated from various ages suggests that chromosome shortening occurs at a rate of ~25 nucleotides per year [172]. To deal with this, chromosome ends are replicated by a crucial reverse transcriptase: telomerase. Progressive shortening of telomeres because of end-replication problems stops cell proliferation and maintains the cells in a metabolically viable, replicative senescence state along with an induced activation of DNA damage checkpoints generated from short telomeres. Interestingly, telomerase expression is silenced in most adult somatic tissues except adult stem cells. These mechanisms for replicative senescence are bypassed in cancer cells, suggesting senescence is a potent tumor suppressor pathway. Conversely, the elongation function of telomerase must also be highly regulated to prevent uncontrolled and deleterious cellular proliferation. Telomeres also function in chromosome positioning within the nucleus by tethering them to the nuclear envelope [173]. *Saccharomyces cerevisiae* has provided groundbreaking concepts in our fundamental understanding of highly conserved telomere biology by its sophisticated genetics in the context of aging and cancer. Besides, the underlying mechanism of DDR (DNA damage response) originating from eroded telomeres is also highly conserved between yeast and higher eukaryotes.

Indeed, phenotypes associated with yeast RLS have been used for the aging of stem cells. Conversely, aging yeast cells do not undergo telomere shortening [174]. Moreover, the genetic manipulation of yeast telomeres length demonstrated an inverse correlation with lifespan [175]. Extended lifespan upon telomerase inactivation is dependent upon SIR protein redistribution on sub-telomeric sites [175]. The RNA and protein component of yeast telomerase is encoded by *TLC1* and *EST2* respectively. Blackburn and colleagues were able to distinguish the phenotypes associated with early vs. late telomerase inactivation (ETI vs. LTI) and showed distinct characteristics of reduced lifespan and telomere senescence [176]. Indeed, ETI in yeast mother cells caused accelerated aging well before telomere attrition and shows characteristics of aging distinct from senescent cells. Indeed, removal of these telomerase components leads to a decrease in lifespan to 7.6 and 12.6 generations respectively, indicating that active telomerase allows normal aging kinetics and alleviates replication stress on telomeres. Moreover, premature yeast mother cell aging by ETI suggests that telomerase loss might lead to telomere-length-independent consequences, causing aging and its related diseases in higher eukaryotes [176].

Moreover, telomerase deficiency in yeast also triggers metabolic alteration, which remains largely unknown. Additionally, there is a strong correlation between telomeric silencing and aging. In yeast, telomere loss does not occur with replicative age but subtelomeric genes are subject to transcriptional silencing [177]. The integration of the URA3 gene at two different telomeres as a function of yeast replicative age revealed a rapid and significant decline with age, raising the possibility that the transcriptional status of subtelomeric genes governs the aging process [178]. Moreover, Sir2, as a component of the heterotrimeric Sir2/3/4 complex, maintains the silencing of both mating-type loci and subtelomeric regions [178]. In humans, a highly intricate signaling mechanism is generated from telomeric alterations in the absence of telomerase, which can have specific effects on the organism level, as can be demonstrated by the acquisition of senescence-associated secretory phenotype (SASP) that can potentially convert senescent fibroblasts into cancer cells [179]. Indeed, there are no reports of SASP or related mechanisms in yeast.

Greider and colleagues [180,181] devised an experiment to measure genome stability upon progressive telomere shortening by depleting telomerase. Yeast telomerase mutants were engineered to carry a dispensable chromosome containing several genetic markers to look for rearrangements upon telomere attrition. They found that prolonged telomere shortening substantially increases (100-fold) chromosomal rearrangements, which could be explained by the activation of a DSB repair mechanism on eroded telomeres. These results were recapitulated in the experimental setting, where yeast cells containing a single short telomere generated by a conditional mutation to diminish telomerase activity were arrested in the G2/M cell cycle phase and showed a bonafide senescence phenotype. Strikingly, these experiments demonstrated that the shortening of a single telomere reduces the number of generations and accelerates cell senescence [182,183]. Indeed, telomere attrition initiates checkpoint activation, as eroded telomeres were found to be enriched for checkpoint factors such as Tel1 and Mec1. Therefore, like in human fibroblasts, checkpoints are activated at the shortest(s) telomere(s) in the cell undergoing senescence, further demonstrating the remarkable conservation in yeast.

Many proteins function in a concerted fashion upon direct or indirect binding to telomeric DNA repeats or sub-telomeric regions and maintain chromosomal integrity by mediating two essential functions: (i) they protect the telomeres from degradation, initiation of DDR and recombination; (ii) they regulate telomere extension via telomerase. In mammals, many nucleoproteins collectively termed as “shelterin” complex (TRF1, TRF2, TIN2, RAP1, POT1, and TPP1) coat the telomeric DNA and protect the chromosome ends. In yeast, the “shelterin” complex constitutes three complexes, Ku70-Ku80 complex, Rap1-Rif1-Rif2 complex, and Cdc13-Stn1-Ten1 (CST) complex, which function cooperatively at telomeric repeats [184]. The DNA damage repair protein Ku is composed of Ku70 and Ku80 heterodimers, which directly associate with telomeres in both mammals and yeast and maintain chromosome integrity. It localizes at the ds-and ssDNA transition zone at telomeres and plays a highly protective role. Ku prevents the degradation of telomeric ends and functionally interacts with the telomerase holoenzyme [185]. Interestingly, the deletion of Ku70-Ku80 leads to early aging phenotype [186] and contributes to cellular senescence [187].

Repressor-activator protein 1 (Rap1) in *Saccharomyces cerevisiae* plays different roles in telomere homeostasis. Rap1 regulates telomere length, inhibits telomere end resection and fusion, and also protects against undesired activation of the DDR checkpoint. Telomeres in budding yeast recruits ~15–20 molecules of Rap1 in a sequence-specific manner and Rif1 and Rif2 bind the Rap1 through its C-terminus and play regulatory roles on telomeres [188]. Rap1/rif1/Rif2 complex binds to dsDNA repeats of telomeres. r*if1∆* and r*if2∆* yeast strains lead to elongated telomeres [189]. Interestingly, in mammals, removal of TRF1, TRF2, and POT1 proteins also result in elongated telomeres [190] indicating a highly conserved negative telomeric regulation.

Cdc13 binds to the telomeric ssDNA and interacts with Stn1 and Ten1 to form the CST complex at the end of telomeres. CST binding at the 3′-overhangs of telomeres renders it inaccessible for telomerase and prevents its replication. CST complex is required for telomere capping and loss of any CST component leads to elongated telomeres and their uncapping [191]. Mutations in these nucleoprotein complexes are associated with a range of pathologies, such as pulmonary fibrosis, aplastic anemia, cardiovascular disorders, and genetic disorders like Coats plus (CP) and dyskeratosis congenita (DC) [192,193]. Understanding the dual mechanism of CST in positive and negative regulation of telomerase could identify therapies for many diseases, including cancer.

## 9. Concluding Remarks

The utilization of yeast RLS as an aging model has paved the way for the identification of pathways affecting longevity in higher eukaryotes. This became possible due to the abundant well-established benefits of yeast in terms of speedy cellular growth, inexpensive growth maintenance, and ease of genetic manipulation, among many other advantages. These advantages provide a clear incentive for the continuous use of the yeast system as an important tool in research on the biology of aging. Yeast aging shares many features which are involved in the response to critical environmental parameters and the level of sensing and conserved responses to nutrient availability and, as an aging model, yeast has been at the forefront of the discovery of Sir2, TOR, RAS, adenylate cyclase, PKA and S6 kinase and the determinants of histone dynamic changes as conserved modulators of longevity.

Moreover, accumulating evidence also indicates that the downstream pathways, such as regulation of stress-responsive transcription factors Msn2/4, Gis1, and Gcn4 and the mechanism underlying reduced translation, enhanced autophagy, control of oxygen radicals, and protective responses have conserved effects on aging in multicellular eukaryotes. Interestingly, the downstream responses of these pathways accounting for delayed aging are highly conserved across eukaryotes. Aging-associated global epigenetic changes raise the possibility of their contribution to several pathologies. Conserved trends of activating and repressive histone marks were reported to be increased and decline respectively and indicate more actively transcribed open chromatin conformations with aging progression. Moreover, aging correlates with the accumulation of DNA damage and the prevalence of H2A.X foci in old as compared to young cells, corresponding to the decreased ability of old cells to repair the damage [194]. Phosphorylated γH2A.X is therefore exclusively present in the damaged regions and helps in the recruitment of repair proteins.

**Figure 5:**
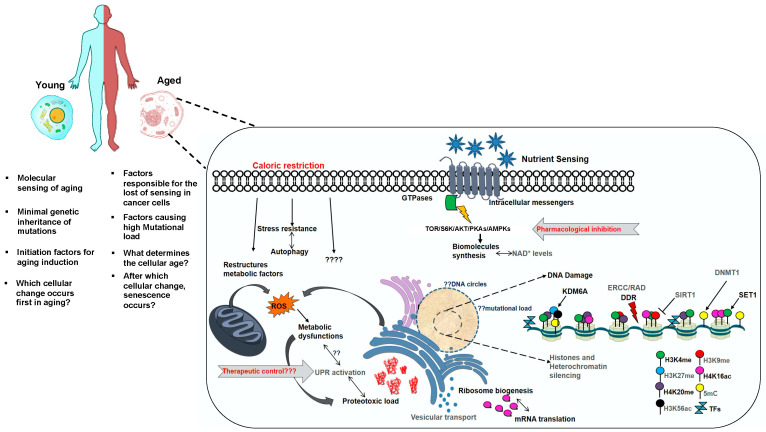
Aging-associated changes occurring at cellular levels. Pathways represented in aged cells in black are up-regulated and severely affect the longevity of organisms, pathways/factors represented in dark grey are down-regulated/non-functional in aged cells. Some of the aging interventions are represented in red color. Question marks in some of the pathways represent obscure feedback/forward mechanisms.

It is evident from many studies that aging brings a plethora of molecular and structural changes that affect the broad cellular physiology of organisms. The interrelations of these changes constitute a complex circuitry to bring about their diverse functions. Many facets of aging are not discussed in this review due to their species-specific effects and some are beyond the scope of the sections included; however, they do contribute towards the overall decline in the regenerative potential of organisms. Some of the unanswered questions remain are the following. How do cells sense aging, and what are the mechanisms which cause cells to learn to die? How are these sensing mechanisms lost in cancer cells to provide them with proliferative potential? How does the mutational load increase in association with aging and what are the underlying drivers? Other aspects of aging-associated features could include feed-back mechanisms that counteract age-related phenotypes. Figure 5 represents the cellular pathways associated with aging and their respective levels in aged cells (refer to the legend of Figure 5).

Data reported to date indicate that yeasts are powerful genetic tools and should, therefore, continue to be exploited as we attempt to understand the biology of human aging and develop therapeutic approaches to mitigate the diseases that accompany it. In addition to the sophisticated pathways described in model organisms, novel studies in emerging models of aging like eusocial insects, and hitherto-uncharacterized aging models, will offer added opportunities for insights into key regulatory pathways of aging. Parallel advancements in high-throughput approaches using broad arrays of models and hypotheses have led to the screening of several molecular and genetic aspects of aging at an “omics” level. The existing databases are tremendously valuable, especially when analyzed collectively. Further efforts are necessary for multi-model integration of data to attain a unified understanding of the development of treatments for aging-related disorders.

## Figures and Tables

**Table 1:** List of some aging-associated genes in yeast and their cellular functions.

Gene	Name Description	Cellular Functions	Impact of Null Phenotype on Lifespan	References
TOR1, SCH9	Ser/Thr protein kinase involved in the signaling of the target of rapamycin	A protein kinase subunit of TOR complex which controls growth in response to nutrients by regulating translation	Increased	[10,40]
SIR2	Silent information regulator	NAD-dependent Histone deacetylase. Plays important roles in silencing at HML, HMR, telomeres, and rDNA	Decreased	[41]
RPL9A, RPL6B, RPL19A	Ribosomal 60S subunit protein L9A	Structural constituent of the large 60S subunit of Ribosome, involved in translation.	Increased	[9,42]
ADH1	Alcohol dehydrogenase enzyme	Reduces acetaldehyde to ethanol during fermentation; involved in NADH oxidation.	Increased	[43]
COX4	Cytochrome *c* oxidase	Subunit IV of cytochrome c oxidase; functions in mitochondrial inner membrane ETC.	Increased	[44]
RPD3	Reduced potassium Dependency	Histone deacetylase, a component of both the Rpd3S and Rpd3L complexes and regulates transcription.	Increased	[45]
DBP3	Dead box protein	RNA-dependent ATPase, involved in rRNA processing.	Decreased	[46]
SGF73	SAGA-associated factor 73	DUB module subunit of SAGA and SLIK; contributes to de-ubiquitination activity.	Increased	[39]
PDE1	Phosphodiesterase	Low-affinity cAMP phosphodiesterase.	Decreased	[47]
SGS1	Slow growth suppressor	ATP-dependent DNA helicase.	Decreased	[48]
FOB1	Fork blocking less	Nucleolar protein that binds to the rDNA replication fork barrier site; required for replication fork blocking.	Increased	[49]
PKH2, HXK1, HXK2	Pkb-kinase homolog	Serine/threonine-protein kinase; involved in signaling cascade; involved in glucose metabolism, endocytosis, and cell wall integrity.	Increased	[50,51]
CDC25	Cell division cycle	Membrane-bound guanine nucleotide exchange factor (GEF); regulates adenylate cyclase.	Increased	[52]
GPR1, GPA2, CYR1	G-Protein-coupled receptor	Senses and integrates nutritional signals and decides cell fate via PKA and cAMP synthesis.	Increased	[53]
PHO84, CIT2	PHOsphate metabolism and CITrate synthase	Effector of retrograde response in the extension of longevity.	Decreased	[54,55]
SOV1	Synthesis of var	Member of the yeast mitochondrial translation control (MTC) module.	Increased	[34]
GCN4, GCN5	General control nonderepressible	Roles in transcriptional activation.	Decreased	[56,57]
ASF1	Anti-silencing function	Role in H3K56 acetylation; involved in chromatin assembly and disassembly.	Decreased	[37]
YPT6	Yeast protein two	Rab family GTPase, required for retrograde transport.	Increased	[58]
YNO1/AIM14	Yeast NADPH oxidase 1/altered inheritance rate of mitochondria	Endoplasmic reticulum localized NADPH oxidase.	Increased	[32]

## Supplementary Materials

The following are available online at https://www.mdpi.com/2218-273X/10/6/882/s1. Figure S1: Structural representation of PKA-inhibitor complex (PDB ID: 4UJ1). Left panel showing a 2D representation of inhibitor 7-[(3S,4R)-4-(3-chlorophenyl) carbonylpyrrolidin-3-yl]-3H-quinazolin-4-one and its interactions to PKA binding-pocket residues (Dark green is showing conventional hydrogen bond, and other residues are participating in different significant interactions). Right panel showing a 3D representation of inhibitor and its interactions to PKA binding-pocket residues. Figure S2: Structural representation of mTOR-inhibitor complex (PDB ID: 4JT5). Left panel showing a 2D representation of inhibitor 2-[4-amino-1-(propan-2-yl)-1H-pyrazolo[3,4-d]pyrimidin-3-yl]-1H-indol-5-ol and its interactions to mTOR binding-pocket residues (Dark green is showing conventional hydrogen bond, and other residues are participating in different significant interactions). Right panel showing a 3D representation of inhibitor and its interactions within PKA binding-pocket. Figure S3: Structural representation of the S6K1-inhibitor complex (PDB ID: 3WE4). Left panel showing a 2D representation of inhibitor 2-{[4-(5-ethylpyrimidin-4-yl) piperazin-1-yl] methyl}-5-(trifluoromethyl)-1H-benzimidazole and its interactions to S6K1 binding-pocket residues (Dark green is showing conventional hydrogen bond, and other residues are participating in different significant interactions). Right panel showing a 3D representation of inhibitor and its interactions to PKA binding-pocket residues.
