# Supplementary material for: Insights into the Conserved Regulatory Mechanisms of Human and Yeast Aging
Source: Biomolecules. 2020 Jun 9;10(6):882. doi: 10.3390/biom10060882 (PMC7355435; doi:10.3390/biom10060882)
Supplement: Supplementary file 1 [file biomolecules-10-00882-s001.pdf]

# Evolutionary connections in human and yeast aging: Mechanism and therapeutic targeting

**Rashmi Dahiya[1,*], Taj Mohammad[1], Mohamed F. Alajmi[2], Md. Tabish Rehman[2], Gulam Mustafa Hasan[3], Afzal Hussain[2], and Md. Imtaiyaz Hassan[1,*]**

[1]Centre for Interdisciplinary Research in Basic Sciences, Jamia Millia Islamia, New Delhi-110025, INDIA

[2]Department of Pharmacognosy College of Pharmacy, King Saud University, Riyadh, Saudi Arabia.

[3] Department of Biochemistry, College of Medicine, Prince Sattam Bin Abdulaziz University, P.O. Box 173, Al-Kharj – 11942, Kingdom of Saudi Arabia.

***Corresponding Author**

**Md. Imtaiyaz Hassan, Ph.D., FRSB., FRSC.**

Centre for Interdisciplinary Research in Basic Sciences
Jamia Millia Islamia, Jamia Nagar
New Delhi 110025, India
E-mail: mihassan@jmi.ac.in

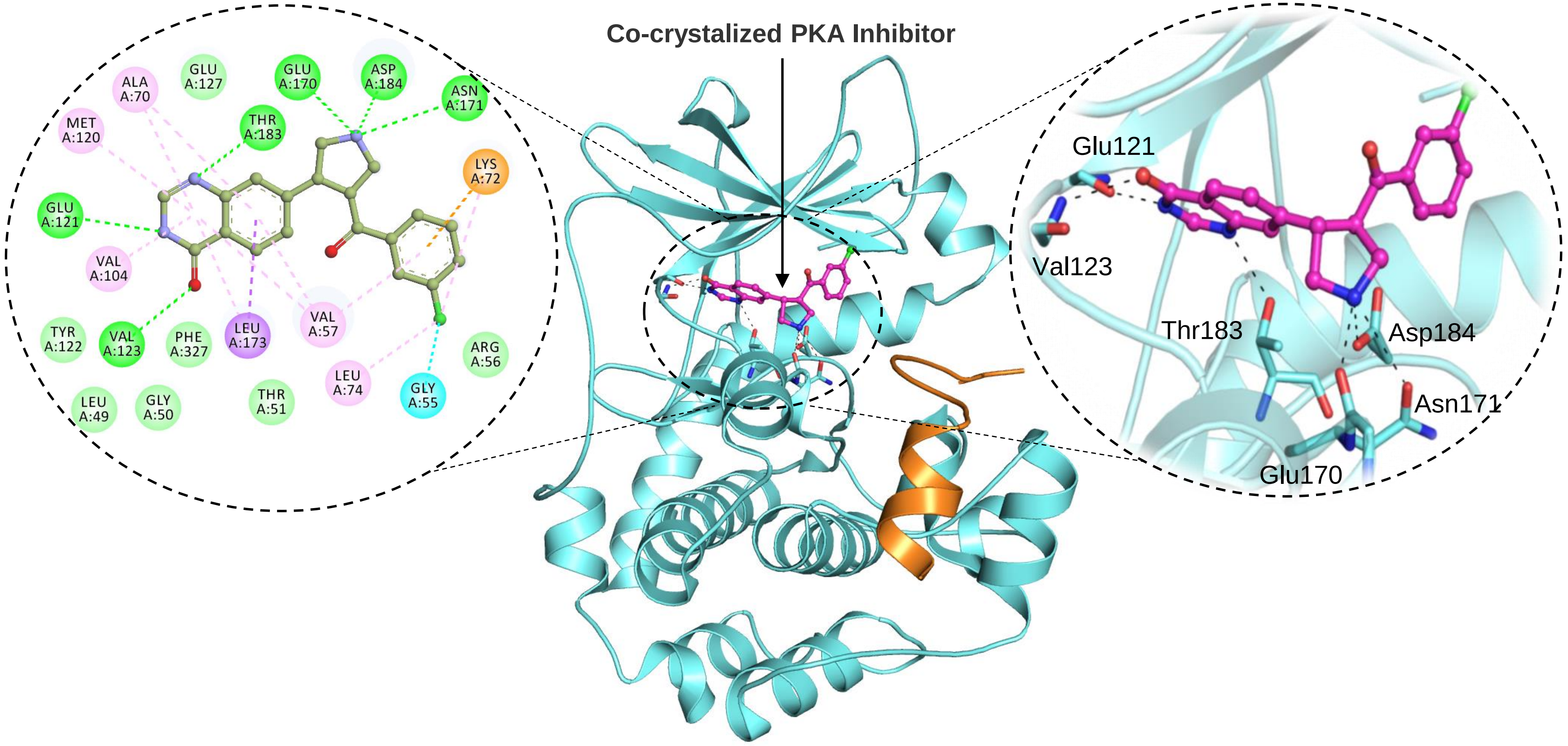


**Figure S1: Structural representation of PKA-inhibitor complex** (PDB ID: 4UJ1). Left panel showing 2D representation of inhibitor 7-[(3S,4R)-4-(3-chlorophenyl)carbonylpyrrolidin-3-yl]-3H-quinazolin-4-one and its interactions to PKA binding-pocket residues (Dark green is showing conventional hydrogen bond, and other residues are participating in different significant interactions). Right panel showing 3D representation of inhibitor and its interactions to PKA binding-pocket residues.

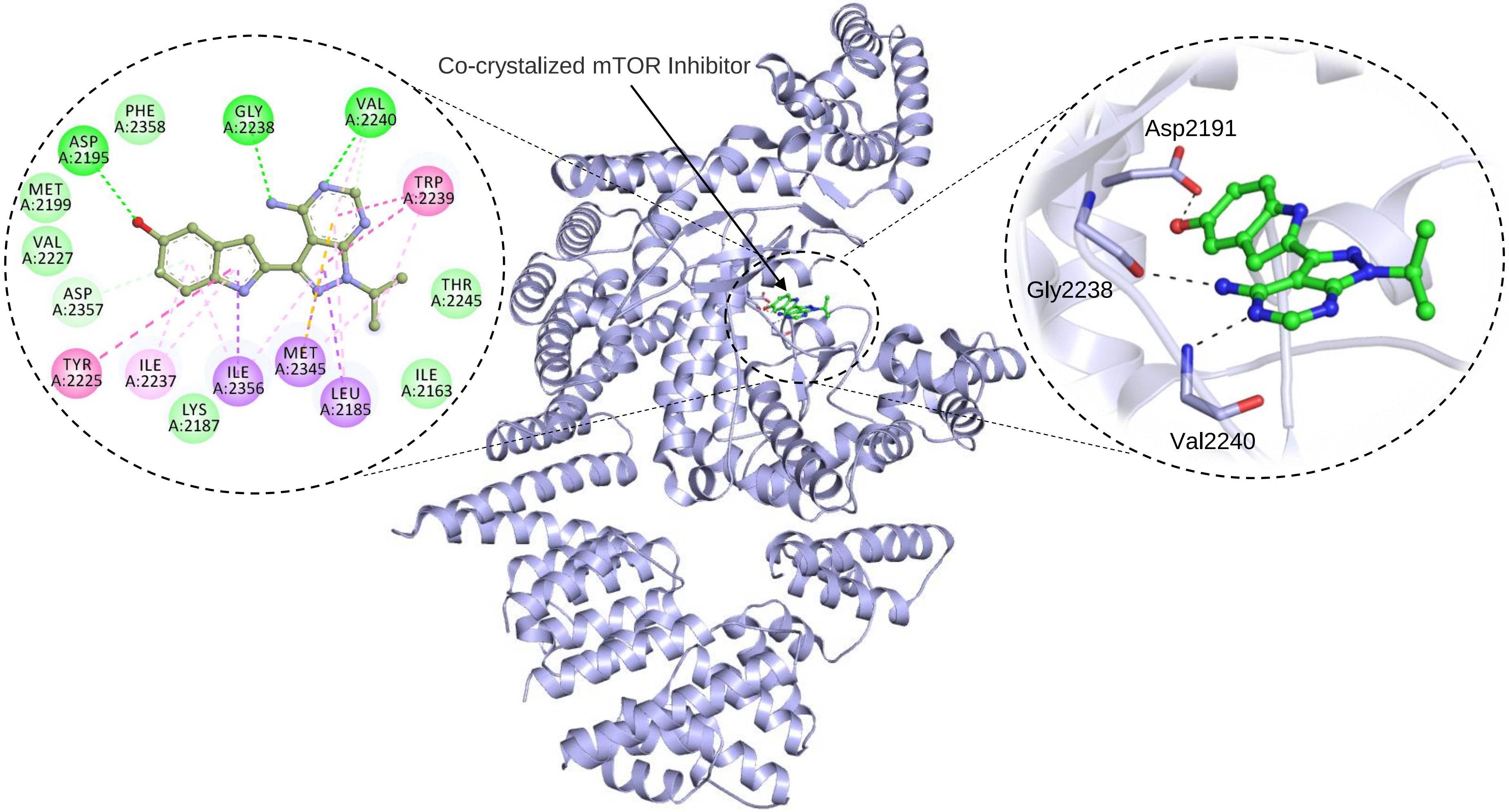


**Figure S2: Structural representation of mTOR-inhibitor complex** (PDB ID: 4JT5). Left panel showing 2D representation of inhibitor 2-[4-amino-1-(propan-2-yl)-1H-pyrazolo[3,4-d]pyrimidin-3-yl]-1H-indol-5-ol and its interactions to mTOR binding-pocket residues (Dark green is showing conventional hydrogen bond, and other residues are participating in different significant interactions). Right panel showing 3D representation of inhibitor and its interactions within PKA binding-pocket.

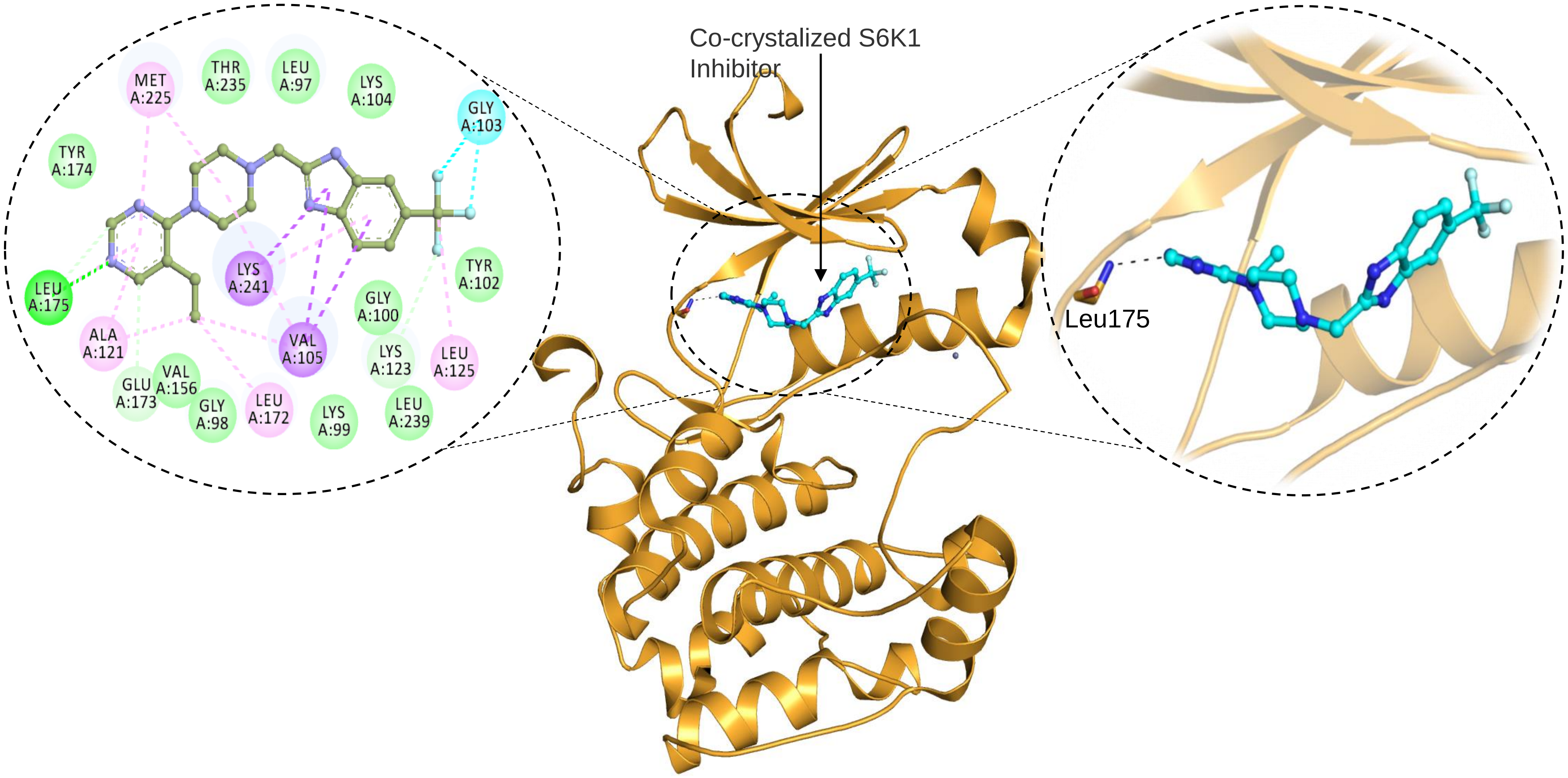


**Figure S3: Structural representation of S6K1-inhibitor complex** (PDB ID: 3WE4). Left panel showing 2D representation of inhibitor 2-{[4-(5-ethylpyrimidin-4-yl) piperazin-1-yl] methyl}-5-(trifluoromethyl)-1H-benzimidazole and its interactions to S6K1 binding-pocket residues (Dark green is showing conventional hydrogen bond, and other residues are participating in different significant interactions). Right panel showing 3D representation of inhibitor and its interactions to PKA binding-pocket residues.
